# Factors of family impact in a Swedish–German cohort of children born with esophageal atresia

**DOI:** 10.1186/s13023-022-02361-2

**Published:** 2022-05-21

**Authors:** Michaela Dellenmark-Blom, Kate Abrahamsson, Jens Dingemann, Stefanie Witt, Carmen Dingemann, Linus Jönsson, Vladimir Gatzinsky, Monika Bullinger, Benno M. Ure, John E. Chaplin, Julia H. Quitmann

**Affiliations:** 1 Department of Pediatrics, Institute of Clinical Sciences, Gothenburg University, The Queen Silvia Children’s Hospital, 416 85 Gothenburg, Sweden; 2 Department of Pediatric Surgery, Queen Silvia Children’s Hospital, Sahlgrenska University Hospital, 416 85 Gothenburg, Sweden; 3 Center of Pediatric Surgery, Hannover Medical School and Bult Children’s Hospital, Carl-Neuberg-Straße, 130625 Hannover, Germany; 4 Department of Medical Psychology, University Medical Center Hamburg-Eppendorf, Martinistraße 52, 20246 Hamburg, Germany

**Keywords:** Esophageal atresia, Congenital malformation, Family functioning, Caregiver

## Abstract

**Background:**

After repair of esophageal atresia (EA), childhood survivors commonly present with digestive and respiratory morbidity, and around 55% have associated anomalies. Although it is known that these problems can reduce health-related quality of life in children with EA, less is understood about the impact on the family. We aimed to identify factors related to family impact in children with EA.

**Methods:**

One parent each of a child with EA (2–18 years) in 180 families from Sweden and Germany answered the PedsQL™ Family Impact Module as the dependent variable. The independent variables were the child’s parent-reported health-related quality of life as measured by PedsQL™ 4.0, current symptoms, school situation, and parent/family characteristics together with child clinical data from the medical records.

**Results:**

Stepwise multivariable regression analysis showed a multifactorial model of the total family impact scores (*R*^2^ = 0.60), with independent factors being the child’s overall generic health-related quality of life, school-absence ≥ 1/month, severe tracheomalacia, a family receiving carer’s allowance, and a parent with no university/college education, *p* < 0.05. Logistic regression analysis showed that an increased number of symptoms in the child the preceding 4 weeks lowered the family impact scores; however, the child’s feeding (*R*^2^ = 0.35) and digestive symptoms (*R*^2^ = 0.25) explained more in the variation of scores than the child’s respiratory symptoms (*R*^2^ = 0.09), *p* < 0.0001.

**Conclusions:**

Family functioning may be a contributing factor to the maintenance of child health. The study findings suggest multifactorial explanations to family impact in children with EA, which are essential when optimizing the support to these families in clinical and psychosocial practice. Future research should explore experiences of family impact from all family members’ perspectives and multicenter studies are warranted to understand better the effectiveness of psychosocial-educational interventions to families of children with EA.

## Background

Esophageal atresia (EA) refers to a discontinuity of the esophagus at birth and occurs in 2.4 of 10.000 live births [1]. Most infants undergo esophageal repair within their first days of life, and if present, closure of a tracheoesophageal fistula. Current survival rates of the children exceed 90%, and the focus of research has become the EA survivors’ long-term morbidities [2] and health-related quality of life (HRQOL) [3]. After repair of EA, children commonly present with dysphagia (43–71%) [4], anastomotic strictures with a need for esophageal dilation (58%), and gastrointestinal reflux disease (44–65%) with management by antireflux medication and/or antireflux surgery [4–6]. Feeding difficulties are present in 63% of the children and can include choking episodes and taking a long time to finish a meal [7]. Symptoms from the respiratory tract are also frequent (52–69%), including, e.g., wheeze, chronic cough, dyspnea, and recurrent airway infections [8, 9]. Although it is known that these problems can reduce HRQOL in children with EA [3], less is still understood about the impact of EA on the family. When the rights of a child living with a rare disease have been set in the context of the United Nations Convention on the Rights of the Child (UNCRC), it was concluded that multiple articles point to the right to advocacy and support for both the parents and the child [10]. Current guidelines for follow-up care from expert networks such as European reference networks for rare inherited and congenital anomalies (ERNICA) and The European and North American Society for Paediatric Gastroenterology Hepatology and Nutrition (ESPGHAN-NASPGHAN) illustrate that there is a need to give more attention on how to care for the families [11, 12] .

The caregiver’s response to their child’s physical and psychosocial needs is described as an essential factor contributing to the child’s psychological development and health [13]. Becoming a parent to an infant with long-term healthcare needs is associated with elevated stress levels, which can increase further if the task of parenthood becomes more demanding [14, 15]. Parental stress concerning pediatric chronic conditions is likely to occur at the time of the child’s diagnosis firstly. The initial diagnosis is followed by a time of reaction to the diagnosis. Last, the parents will experience an adjustment period, including adjusting to managing the child’s condition and providing their child with long-term support [16]. While the increased emotional burden is consistently reported by parents of children with chronic health conditions [14, 15], we also know that parents of a child with a rare disease additionally may experience a feeling of a lack of competence regarding the diagnosis, poor contact with health care providers, lack of social connection with families of children with similar conditions with subsequent feelings of loneliness and a financial burden [17]. The indefinite uncertainty related to a rare disease may impede active coping, seeking instrumental social support, and positive reinterpretation and growth, hence hindering a good adaptational process [18].

In families of children with EA, most studies have found a high parental burden resulting in clinically significant stress disorders [19, 20], depression [21, 22], impaired mental quality of life [23], and parental experiences of physical and social restrictions [24]. Risk factors of impaired mental health in parents of children with EA are being a mother vs. father of a child with EA, having lower versus higher family income, and being a parent of a 2–7-year-old versus 8–17-year-old child with EA [23]. Two studies of families with children with EA have shown that the factors that are associated with poor family functioning are having a child with feeding problems and associated anomalies [24] or emotional and behavioral problems [25]. The weakness in most studies in this area is that they use a single-center design with small sample sizes with less than 50 children [19–21, 23, 25, 26]. No study has addressed a holistic explanation model to understand which factors are most significant to the EA children’s family impact. When the family is functioning well, the parents will be better positioned to manage their child’s health care needs and appointments and support their development [16]. Given this background, this study aimed to take a broad approach and identify clinical and psychosocial factors of family impact in a Swedish–German cohort of children born with EA. We hypothesized that clinical variables indicating disease severity as well as psychosocial factors would impact these children’s families.

## Methods

This study was part of an international project to address the needs of care and improve knowledge of HRQOL among children with EA and their parents. Study approval was obtained from the local ethics committees of Gothenburg, Sweden (958-13) and Hannover, Germany (2936-2015).

### Participants

Children (2–18 years old) born with EA, anatomical subtypes A–E [27], were identified in the hospital databases of the Queen Silvia Children’s Hospital in Gothenburg, Sweden, (n = 141) and the Center of Pediatric Surgery, Hannover Medical School and the Bult Children’s Hospital, Hannover, Germany (n = 105). All patients and one of their parents were invited to participate in the study during 2016. Ability to understand Swedish or German and written informed consent from legal guardians and children ≥ 15 years were required for study participation.

### Data collection

#### Clinical information

At each clinical center, a researcher reviewed the children’s medical records to retrieve information of birth characteristics, the anatomical subtype of EA according to the Gross classification system [27], associated anomalies, and surgical interventions for participants and non-participants.

#### Measures

The questionnaires were sent to the families by post for completion at home and for return using a pre-paid envelope. A maximum of three reminders were sent to non-respondents to increase the response rate.

##### Parent/family characteristics, current child health, and school situation

One parent from each family answered a standardized questionnaire developed by the authors, including 20 questions of sociodemographic and socioeconomic information of the parents, data on the child’s health the previous 4 weeks covering occurrence of feeding, digestive and respiratory problems, and data on the child’s school situation.

##### Generic HRQOL

The parent completed a validated generic and a condition-specific questionnaire to assess their child’s HRQOL [28, 29]. In this study, we used the parent-proxy-reported answers of the children’s generic HRQOL as measured by PedsQL™ 4.0 generic core scales (PedsQL™ 4.0) for healthy children and children with chronic conditions because it enables a total score calculation for the age group 2–18 years [29]. While the version for children 2–4 years old comprises 21 items, the versions for children aged 5–7, 8–12, and 13–18 years old include 23 items, but all age-specific versions of PedsQL™ 4.0 measure physical (8 items), emotional (5 items), social (5 items), and school functioning (5 or 3 items) in the past 4 weeks. Each item is scored on a 5-point Likert scale (never a problem to always a problem).

##### Family impact

**Fig. 1:**
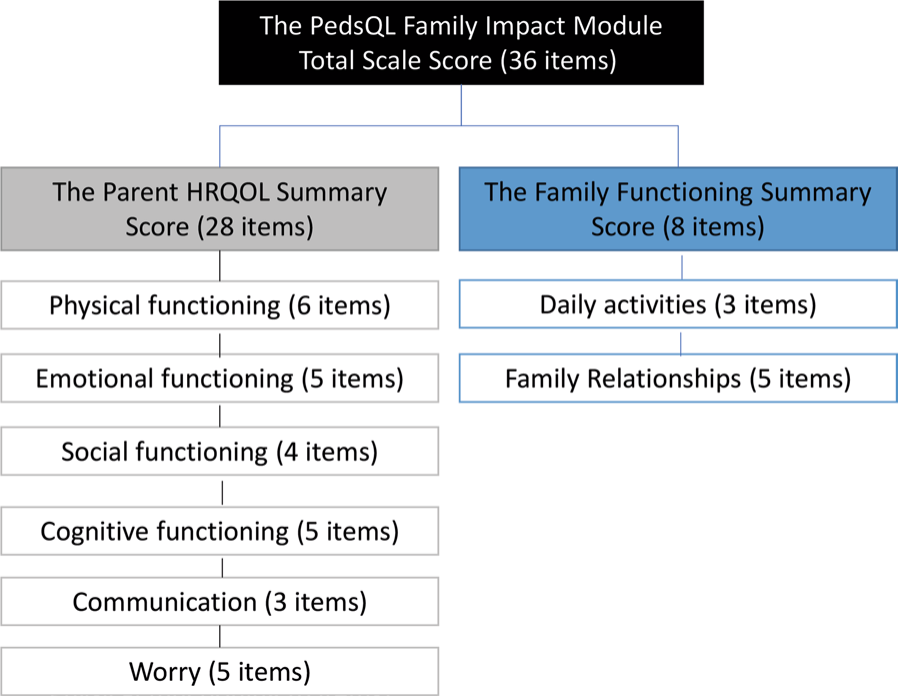
The scale structure of the 36-item PedsQL™ family impact module, which is designed to assess the impact of pediatric chronic health conditions on the family

The parent also answered the 36-item PedsQL™ Family Impact Module, a validated instrument designed to assess the impact of pediatric chronic health conditions on the family in the last 4 weeks [30]. As viewed in Fig. 1, the instrument comprises the eight scales of physical functioning, emotional functioning, social functioning, cognitive functioning, communication, worry, daily activities, and family relationships. Each item is scored on a 5-point Likert scale (never a problem to always a problem). The PedsQL™ Family Impact Module yields an overall score of family impact, The PedsQL™ Family impact module total scale score.

### Data analysis

Data were analyzed using IBM SPSS Statistics for Windows (version 23.0, Armonk, NY, USA: IBM Corp) and SAS 9.4 (SAS Institute Inc., Cary, NC, USA). Mean values and standard deviations were calculated for continuous variables, and frequencies and percentages were calculated for categorical variables. In the PedsQL™ 4.0 [31] and the PedsQL™ Family Impact Module [30], parents’ responses to the 5-point Likert scale were transformed to a 0–100 scale, with higher scores denoting better child HRQOL and less family impact (i.e., better family functioning), respectively.

The method included analyzing the influence of *clinical factors* (congenital, pediatric surgical, and current symptom-related) and *psychosocial factors* (the child’s or parent’s psychological or social status) on the PedsQL™ Family Impact Module Total Scale Score. Using regression analysis, the clinical and psychosocial factors (further detailed in Table 1) were treated as possible independent variables and The PedsQL™ family impact module total scale score [30] as the dependent variable. First, univariable regression analysis was performed to identify congenital, pediatric surgical, and psychosocial factors, which explained a proportion of the variance in the PedsQL™ family impact module total scale score, presenting intercept/*β*_*O*_ (constant of each Summary Score), Beta/*β* (estimate of variation of scores) with 95% confidence interval (CI), *p* value and *R*^2^ for each factor. Next, the variables with *p* < 0.1 in the univariable analysis were included in the stepwise multivariable regression analysis to identify *independent* factors influencing The PedsQL™ family impact module total scale score. The significant level was *p* < 0.05.

**Table 1:** Study population characteristics and descriptives of PedsQL™ family impact module

Child clinical and psychosocial characteristics	*n*_replies_	*n* (%)	Mean (SD)
*Congenital characteristics*			
Prematurity, gestational age < 37 weeks	171	67 (39)	
Low birth weight, birth weight < 2500 g	169	67 (40)	
Child gender male	180	72 (40)	
Long-gap esophageal atresia/esophageal atresia, gross type A and B^a^	180	24 (13)	
Associated anomalies	180	106 (59)	
Cardiovascular malformation	180	48 (27)	
Anorectal malformation	180	20 (11)	
VACTERL association^b^	180	26 (14)	
Severe tracheomalacia^c^	180	27 (15)	
*Pediatric surgical characteristics*			
No primary esophageal anastomosis	180	24 (13)	
Gastrostomy insertion		56 (32)	
Revisional surgery following repair due to anastomotic leak or recurrent fistula	180	25 (14)	
Number of esophageal dilatations	178		3 (11)
Severity level of esophageal atresia			
Severe esophageal atresia^d^	180	95 (53)	
*Child psychosocial characteristics*			
Child receiving additional school support	161	56 (35)	
Child with high school absence, ≥ 1/month	180	38 (24)	
Parent-reported PedsQL^™^ 4.0 total scores	180		79.2 (19.0)
Child age	180		9.3 (4.7)
*Parent/family characteristics*			
Mother	180	159 (88)	
Single parent	175	26 (15)	
Parent having no college or university education	178	99 (56)	
Doctor-diagnosed parental disease	177	29 (16)	
Parent on sick leave the previous year	172	9 (5)	
Family receiving financial carer allowance previous year	172	54 (31)	
Family residence in Germany	180	56 (31)	
Family resident in rural area	176	46 (26)	
*The PedsQL™ family impact module scale score (parent-report)*			
Family impact module total scale score	180		75.2 (19.4)

^a^Pure esophageal fistula (Gross A) or Esophageal fistula with a proximal tracheoesophageal fistula (Gross B)

^b^Requires at least three anomalies of vertebral defects, anal atresia, cardiac defects, tracheoesophageal fistula, renal anomalies, and limb abnormalities

^c^Severe tracheomalacia/tracheobronchomalacia, verified through flexible bronchoscopy to have an anteroposterior collapse during coughing and expiration documented as ≥ 75%, excessive and/or severe

^d^At least one of the following criteria: primary anastomosis was delayed and/or EA replacement was accomplished, presence of a severe tracheomalacia, presence of at least one other congenital health condition resulting in disability

A priori, we decided to analyze the impact of the child’s current symptoms on The PedsQL™ family impact module total scale score using logistic regression analysis and presenting the number of observations, *β*_*O*,_ *β*_*1*,_ *R*^2^. We analyzed feeding, esophageal and respiratory problems separately because, as in earlier studies [28], we wanted to analyze the symptom burden given by its main characteristics. The hypothesis was that an increased number of different symptoms in the preceding 4 weeks would lower The PedsQL™ family impact module total scale score. The significant level was *p* < 0.05.

## Results

### Study sample

Of all 246 invited families, 180 families accepted the study invitation, returned informed consent, and completed the PedsQL™ family impact module (124/141 families from Sweden, 56/105 families from Germany). Table 1 presents children and parents’ clinical and psychosocial characteristics, which were also used in the regression analysis as possible independent variables of the PedsQL™ Family Impact Module Total Scale Score. As detailed in Tables 1 and 72 (40%) of the children were male, 24 (13%) were born with long-gap EA, 106 (59%) had associated anomalies and 25 (14%) had major revisional surgery after repair of EA. At follow-up, 56 (35%) of the children had additional school support and 38 (24%) school absence ≥ 1/month the past year. The vast majority of parent-proxies were mothers (*n* = 159, 88%). Of the parent-proxies, 99 (56%) had no college or university education, 29 (16%) had a doctor-diagnosed disease, 9 (5%) were on sick leave in the past year, and 54 (31%) received financial carer’s allowance. No significant differences in the presented clinical child characteristics (Table 1) were identified between the study sample and non-participants or between study participants from Sweden and Germany.

### Impact of congenital, pediatric surgical, and psychosocial factors on the family

#### Results of the univariable regression analysis

**Fig. 2:**
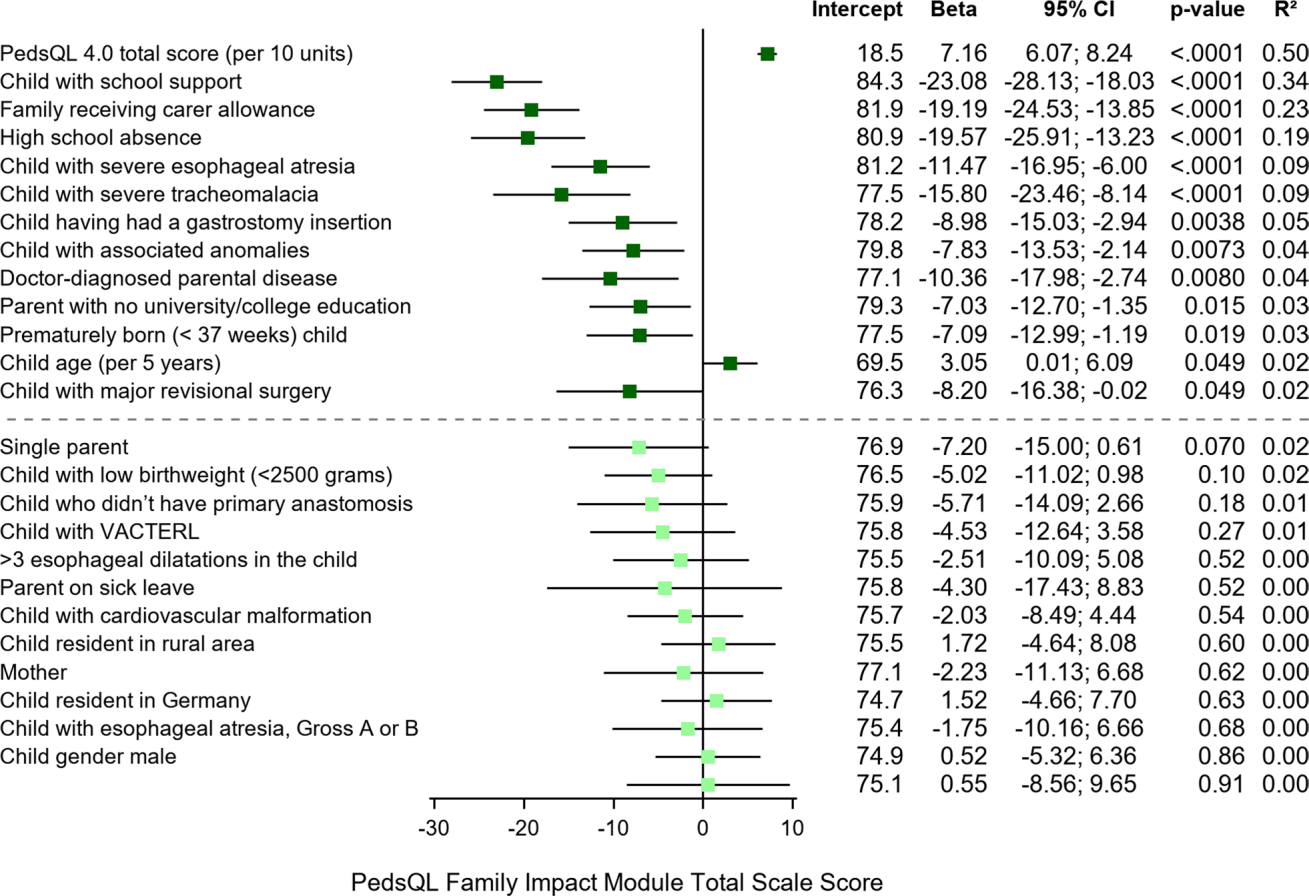
The results of the univariable regression analysis displaying factors potentially influencing the PedsQL family impact module total scale score (0–100), showing intercept/constant, β with 95% confidence interval (CI), *p* value, and *R*^2^. The significance level was *p* < 0.05. Factors being significant are listed above the dashed line. Factors associated with a negative relationship with the PedsQL Family impact module total scale score are visible with the square on the left side of 0 and those with a positive relationship on the right side of 0

The results of the univariable regression analysis in investigating factors potentially influencing the PedsQL™ family impact module total scale score are detailed in Fig. 2. As shown, eleven factors were negatively associated with the PedsQL™ Family impact module total scale score (*p* < 0.05). Three of these were congenital factors (prematurity, associated anomalies, severe tracheomalacia), two were pediatric surgical factors (history of gastrostomy insertion, major revisional surgery following esophageal repair), and one categorization of the child having severe EA, all of which indicated clinical disease severity of the child with EA. Moreover, five factors were psychosocial factors (child with school support, high school absence, a family receiving carer’s allowance, a parent having a doctor-diagnosed disease, and with a parent with no university/college education). Two psychosocial factors, namely the child’s overall generic HRQOL and child age (every 5 years), were *positively* associated with the total PedsQL™ Family impact module total scale score (*p* < 0.05). Three factors explained > 20% in the variation of the total PedsQL™ family impact module total scale score, namely the child’s overall generic HRQOL (PedsQL™ 4.0 total scores) according to parent-proxy report (*R*^2^ = 0.50), a child with additional school support (*R*^2^ = 0.34), and a family receiving carer’s allowance the past year (*R*^2^ = 0.23), *p* < 0.0001.

#### Results of the multivariable stepwise regression analysis

Table 2 presents the stepwise multivariable regression analysis results, showing the *independent* factors influencing the PedsQL™ family impact module total scale score. The multifactorial model of five factors (*R*^2^ = 0.60) showed that as generic HRQOL scores in children with EA increased, so did the PedsQL™ family impact module total scale score as rated by the parent. In contrast, a child’s high school absence, severe tracheomalacia, a family receiving a carer’s allowance, and a parent with no university/college education were associated with a decrease in the PedsQL™ family impact module total scale score, *p* < 0.05.

**Table 2:** Independent factors influencing the PedsQL family impact module total score in children with esophageal atresia

PedsQL family impact module total score	43.8 (*β*_*O*_)	*p* value
*n* = 152, *R*^2^ = 0.60	*β*_1–5_	
PedsQL 4.0 total score (per 10 units)	5.1	< 0.0001
High school absence, ≥ 1/month	− 11.1	< 0.0001
Child with severe tracheomalacia^a^	− 9.9	0.002
Family receiving carer allowance the past year	− 7.2	0.005
Parent with no university/college education	− 4.5	0.027

Results from stepwise multivariable analysis of factors influencing the PedsQL family impact module total score applied to parents of children born with esophageal atresia

^a^Severe tracheomalacia/tracheobronchomalacia, verified through flexible bronchoscopy to have an anteroposterior collapse during coughing and expiration documented as ≥ 75%, excessive and/or severe

#### Impact of the child’s symptom burden on the family

Table 3 presents the prevalence of digestive symptoms, feeding difficulties, and respiratory symptoms in the past month in the study sample of children with EA. Logistic regression analyses demonstrated that an increased number of different symptoms in the child in the preceding 4 weeks was significantly associated with a lowered PedsQL™ family impact module total scale score. As detailed in Table 4, a higher number of different feeding problems (*n* = 161, *β*_*O*_ = 85.9, *β*_1_ = − 5.3, *R*^2^ = 0.35), a higher number of different digestive problems (*n* = 168, *β*_*O* _ = 87.1, *β*_1_ = − 10.3, *R*^*2*^ = 0.30) and a higher number of different respiratory symptoms were significantly associated with deceased PedsQL™ Family Impact Module Total Scale Score (*n* = 164, *β*_*O*_ = 82.4, *β*_1_ = − 3.4, *R*^*2*^ = 0.09), *p* < 0.0001. Feeding and digestive symptoms explained more in the variation of the total scores than the child’s respiratory symptoms.

**Table 3:** Presence of digestive, feeding and respiratory symptoms the past month in children with esophageal atresia

	n_tot_	n	%
*Digestive symptoms*			
Dysphagia	174	72	41.4
Heartburn	172	64	37.2
Vomiting problems	172	45	26.2
*Feeding difficulties*			
The child needs increased fluid intake during meals	172	79	45.9
The child needs > 30 min to finish a large meal	171	45	26.3
The child needs to avoid certain food	164	42	25.6
The child needs to eat small portions	173	40	23.1
The child needs energy-enriched food	174	33	19.0
The child needs additional assistance by an adult during meals	173	30	17.3
The child needs adjusted food consistency	172	23	13.4
The child needs nutrition through a gastrostomy	173	18	10.4
The child needs nutrition through infusion pump	172	9	5.2
*Respiratory symptoms*			
Cough	173	98	56.6
Breathlessness on physical exertion/at rest	171	65	38.0
Airway infections	172	60	34.9
Wheezing at physical activity/at rest	171	56	32.7
Chest tightness/pain	170	30	17.6

**Table 4:** Relationships between symptoms and PedsQL family impact total scale score in children with esophageal atresia

	Feeding difficulties^a^					Digestive symptoms^b^						Respiratory symptoms^c^				
	*n*	*β*_0_	*β*_1_	*R*^2^	*p* value	*n*	*β*_0_	*β*_1_	*R*^2^	*p* value	*n*		*β*0	*β*1	*R*^2^	*p* value
The PedsQL family impact total score	161	85.9	-5.3	0.35	*p* < 0.0001	168	87.1	− 10.3	0.30	*p* < 0.0001	164		82.4	− 3.4	0.09	*p* < 0.0001

^a^Feeding difficulties (n_max_ = 9) food restriction/eats small portions/energy-enriched food/adjusted food consistency/>30 min to finish a large meal/increased fluid intake to ease swallowing food/food through a gastrostomy/nutritional intake through infusion pump/adult assistance during meals

^b^Digestive symptoms (n_max_ = 3); food impaction/difficulty swallowing food, heartburn, vomiting

^c^Respiratory symptoms (n_max_ = 5); cough, wheezing at physical activity/at rest, airway infections, breathlessness on physical exertion/at rest, chest tightness

## Discussion

This study showed that the associated factors of family impact in children with EA are the child’s level of generic HRQOL, school absence, tracheomalacia, as well as the parental use of carer’s allowance and parent educational level. Furthermore, the study findings suggest that an increased number of feeding, digestive and respiratory problems in children with EA are negatively associated with family impact. Hence, this illustrates multifactorial explanations of the family impact that expand beyond a medical-surgical model.

In the present study, parents’ ratings of their EA child’s generic HRQOL were highly associated with the level of family impact. Already in the univariable analysis, the child’s generic HRQOL explained 50% of the total family impact scores. Following stepwise regression, which is a method of regressing multiple variables and simultaneously removing the weakest correlated variable, parents’ ratings of their child’s generic HRQOL remained a part of the multifactorial model to explain the total family impact scores. The PedsQL™ family impact module total scale score is built up by two subscales measuring parents’ HRQOL and family functioning, with most items targeting parents’ HRQOL. Therefore, the results could mean that children’s and their parents’ HRQOL are interlinked, as reported by parents. In the PedsQL™ 4.0 generic core scales, the parent-proxy should rate how he/she believes the child experiences their HRQOL [29]. Parents of children with chronic conditions tend to rate their child’s generic HRQOL lower than their children [32], including parents of children with EA [33, 34]. However, previous studies have also shown that the child–parent agreement in ratings of the EA child’s HRQOL is mainly good and any discrepancy is not explained by the level of family functioning [34].

Moreover, we found that EA children’s school situation impacted the family. This finding agrees with a small German study, which showed that frequent school absence was associated with impaired mental HRQOL in their parents [23]. In pediatric cyclic vomiting syndrome, the authors also made similar observations. They suggested that children missing school create parental worry, parents missing work, decreased family income, and limits parents’ capacity to attend to other responsibilities [35]. In a Swedish study, school absence ≥ 1/month in children with EA was associated with the child’s use of school support, including educational support, support with nutritional intake, or both [36]. Educational support may be provided to children with EA with emotional and behavioral problems, a group of children previously shown to be at risk for having worse family functioning [25]. Although regulations of children’s right to school support vary between Sweden and Germany, it may imply underlying health care needs of the children, which could explain our findings.

This study showed that the *independent* clinical factor in the multifactorial model explaining family impact was the presence of severe tracheomalacia in children with EA. The relation between severe tracheomalacia (verified through bronchoscopy) and family impact is a new finding to the authors’ knowledge. In our study sample, bronchoscopy was performed preoperatively in theatre or follow-up care of children with severe airway symptomatology. As detailed in Table 1, we standardized criteria to define severe tracheomalacia [37], with the most recent bronchoscopy valid for inclusion. EA children with severe tracheomalacia are a disease subgroup with a risk for respiratory and gastroesophageal dysmotility problems [8]. Our subsequent analysis showed that several feeding and airway problems were significantly more prevalent in EA children with severe tracheomalacia than those without. Similarly, this group used more antireflux medication, bronchodilators or inhaled steroids. Other clinical variables indicating disease severity, such as associated anomalies, premature birth, and a child with a history of gastrostomy feeding, were significantly associated with lower family impact scores in the univariable analysis. However, after the multivariable regression analysis, these clinical variables were not retained as a part of the multifactorial model. Previous research has shown that associated anomalies in children with EA negatively impact family functioning [24] while parents of children with VACTERL association (i.e., with at least three of the following: vertebral defects, anal atresia, cardiac defects, tracheoesophageal fistula, renal anomalies, limb abnormalities) self-report levels of anxiety and depression comparable to non-clinical samples [38].

In this study, 31% of parents received carer’s allowance, which corresponded to poor family functioning, probably because carer’s allowance is provided to parents of children with severe healthcare needs. Furthermore, 56% of parents had no college/university degree, a factor that is consistent with previous studies in corresponding to lower parental HRQOL [39]. In contrast to other studies of parents’ mental health and HRQOL [14], including studies of rare diseases [23, 40], being a mother was not a risk factor for negative impact on the family, but similar to previous studies [41], mothers represented the majority of proxy-reports of children. Perhaps the caregiver burden and limitation in career development add to the explanation of a participant’s perceived family impact. Moreover, child age was significant in the univariable regression analysis but was not an independent factor of family impact in children with EA. In previous studies, the parents’ psychological burden is less in older children with EA than younger children [19, 23, 26], which is reasonable as the child’s health may improve with increased age [42]. A reason for our results may be the broader set of variables included in the multivariable stepwise regression analysis.

Lastly, we investigated the impact of the child’s symptoms on the family. While feeding disorders have been shown to negatively impact families of children with EA [24], this study adds that the child’s feeding and digestive problems are explained substantially more in the variation of the total family impact scores than the child’s respiratory problems. This is congruent with factors influencing EA children’s condition-specific HRQOL [28]. Healthy newborn infants learn to suck competently within days after birth and develop a feeding-sleeping cycle in the context of a caregiver relationship [43]. Following the repair of EA, children may need a prolonged time to establish peroral feeding compared to healthy infants, with a need for gavage feeding or even gastrostomy feeding for a time. In a previous study of maternal-infant social interaction, the most significant area of concern for mothers of children with EA was during feedings [44]. The child’s experiences of vomiting, choking, and food impaction may interact with the child’s ability and willingness to eat. From a parent’s perspective, the frequent daily attempts at trying to feed a child with feeding difficulties can be tiring and impact family mealtimes [43]. This context could help to explain our study results.

### Implications of the study findings

Knowledge of risk factors for poor family functioning in children with EA provides essential information for health care professionals and patient advocacy groups encountering these families in order to understand which families may need targeted support. Some families of children with EA can adjust and develop a fulfilling life after encountering a medically challenging life event. Others are likely to develop worry, stress, or depressive symptoms in the long term [22, 45]. Our study findings would stress the importance of holistic caring support to optimize EA child and family health, especially since it has been shown that there is ample room for improvement to facilitate the pathways to psychosocial care for children with rare diseases and their families [46]. First, since the level of generic HRQOL in children with EA was related to family impact, communication of the child’s HRQOL in a clinician-family encounter during follow-up care with subsequent efforts to strengthen the child’s HRQOL could be of importance to the child [47], but also to the whole family. Then, because a higher symptom burden in children with EA was associated with worse family impact, this emphasizes the need for multidisciplinary follow-up care managed by doctors, pediatric nurses, dieticians, speech therapists, psychologists, and social workers. This team can help monitor and treat the child’s symptoms properly, but also provide practical and educational support to families of children with EA and facilitate a good adaptational process. This would be in line with patient-driven health-care recommendations for adults with EA and their families [48]. Moreover, since our study results imply that family impact in children with digestive symptoms and feeding difficulties is worse, these families may benefit from having targeted support early after the child is born with EA. In this perspective it has been pointed out that early intervention on feeding issues in infancy may reduce later problems [49], and that mothers of children with EA may mourn the loss of their initial fundamental role as feeders of their child [43]. A possible support to these families may therefore be a health care professional and/or a patient advocacy group to listen to the parents, allow them to verbalize their feelings about feedings of their child and reinforce adaptation to some of their caregiver roles.

### Study strengths and weaknesses

Although EA is rare, numerous children worldwide are affected, and specialized knowledge is desirable to help them and their families. To the authors’ knowledge, our study represents the only international study to focus on the family impact in children with EA. We had a high number of respondents compared to previous literature within the field that investigated parent och family impact [19, 23–25]. Congenital characteristics (prematurity, low birth weight, anatomical subtype of EA, associated anomalies) and frequency of digestive, feeding, and respiratory problems agree with the previous literature [2]. This suggests the generalizability of the study findings. However, as the PedsQL™ Family Impact Module [30] asks questions regarding problems due to the child’s chronic condition, this study focused on negative impacts. Comparing family impact with healthy peers was inappropriate, and a sibling- or child/self-report was not applicable. We did not use a standardized, validated score assessment of the parents’ socioeconomic standards or clinical symptoms since appropriate ones were not available in Swedish and German at the time of the study. Nevertheless, this limits the study findings’ generalizability. We did not detail what additional school support included, and the cognitive ability of children EA may vary since cognitive dysfunction was not an exclusion criterion for our study. We measured the proportion of parents with a doctor-diagnosed disease and parents on sick leave the last year. However, details are not further presented in this article because of personal integrity reasons. Of note, we did not systematically study the educational level of the non-responding parent in the family, nor his/her perception of family impact. In pediatric research, the child’s self-report of HRQOL is of primary importance, but we used the parent-proxy-report because it enables score calculations from child age 2–18 years. The study setting was two North European countries with similar and different societal conditions for families of children with EA. Although the overall response rate was acceptable, missing data may weaken the regression analysis. The relationship of many possible factors influencing the total family impact score was tested, increasing the risk of Type 1 error. However, stepwise regression was used to remove the weakest correlated variable.

## Conclusions

The independent factors of family impact in children with EA are the child’s level of generic HRQOL, school absence, tracheomalacia, as well as the parental use of carer’s allowance and parent educational level. Furthermore, feeding and digestive problems in children with EA are prominently and negatively related to the level of family impact. The study findings are helpful for clinical and psychosocial practice and patient advocacy groups of EA when developing family-centered support. Future research should explore experiences of family impact, adaptation, and health care from the mothers’ and fathers’ perspectives using a qualitative approach as well as the experiences of being a sibling to a child with EA. Multicenter studies are warranted to understand better the effectiveness of psychosocial-educational interventions to families of children with EA.

## Data Availability

The datasets analyzed during the current study are available in the manuscript. Further information in earlier publication or is not available in public due to lack of ethical approval.
